# Management of Enterocutaneous Fistula in Crohn’s Disease by Embolization With Glue Injection and Coiling: A Case Report

**DOI:** 10.7759/cureus.43089

**Published:** 2023-08-07

**Authors:** Sara Shbaita, Laith Daraghmeh, Nael Abu Saleem, Alaa Rostom, Qusay Abdoh, Iyad Maqboul

**Affiliations:** 1 Faculty of Medicine, An-Najah National University, Nablus, PSE; 2 General Surgery, An-Najah National University Hospital, Nablus, PSE; 3 Radiology, An-Najah National University Hospital, Nablus, PSE; 4 Gastroenterology and Hepatology, An-Najah National University Hospital, Nablus, PSE

**Keywords:** case report, coiling, fibrin glue, embolization, crohn's disease, enterocutaneous fistula

## Abstract

There is one reported case of a pancreatoduodenal fistula that was managed using combined coil embolization and fibrin glue after the failure of other methods. Herein, we document this case to highlight the value of coil embolization and fibrin glue as surgical alternatives for fistula treatment. We present a case of a 39-year-old female patient who has a known case of Crohn’s disease (CD) and presented with an enterocutaneous fistula (ECF) after her most recent surgery. With the failure of conservative approaches and as she refused any surgical interventions, fibrin glue injection and coiling were used. As a conclusion, embolization may work well as a surgical management alternative due to its simplicity.

## Introduction

An enterocutaneous fistula (ECF) is an uncommon yet debilitating complication that can arise from a variety of underlying diseases [1]. Inflammatory bowel diseases (IBDs), most notably Crohn's disease (CD), are among the potential causes of fistula formation with poorly defined pathophysiology [2]. CD-associated fistula has been shown to arise either in diseased bowels, consistent with the active disease process, or at related surgically created anastomosis sites [3]. This complication is associated with considerable morbidity and mortality rates owing to subsequent metabolic abnormalities, sepsis, and malnutrition. Furthermore, the remarkable recurrence rate has been proven to be high in recent years, with a higher rate in the presence of an inflammatory bowel process [4]. The management of ECFs is challenging and directed toward controlling associated comorbidities of sepsis, malnutrition, and fluid and electrolyte abnormalities, with surgery remaining the mainstay of definitive therapy [5], as most fistulas that fail to close within four weeks with conservative management will need surgical interventions [6]. Unsurprisingly, fistulas formed in the setting of CD pose a higher management challenge and urge the need for a multidisciplinary approach [7].

Fibrin glue has been utilized in the treatment of fistulizing CD to avoid the necessity for surgical interventions [8]. The literature is abundant in its application in treating perianal fistulas but lacking in its effect on ECFs. Coiling has also been employed and has demonstrated good outcomes in treating persistent ECFs despite surgical interventions [9]. In this case, we describe a female patient with a history of CD who presented complaining of abdominal pain, fever, and surgical site discharge several months after an ileocecectomy and ileocolic anastomosis procedure. Further examination revealed abscesses and an ECF extending from the anastomosis site to the surgical wound. She was a suitable candidate for embolization because she declined any surgical intervention. The patient was admitted to a non-profit medical and academic facility and was later treated with fibrin glue injections and coiling.

## Case presentation

A 39-year-old female patient was brought to the emergency department (ED) by her family with complaints of generalized mild abdominal pain, frequent episodes of vomiting, subjective fever, and increasing discharge from a surgical wound. Her previous surgical history was significant for right hemicolectomy with end-to-end ileocolic anastomosis following iatrogenic bowel perforation six months prior to this admission.

Her past medical history revealed a 10-year history of CD, which was partially controlled by mesalamine and 5-aminosalicylic acid (5-ASA) throughout, with multiple exacerbation attacks managed conservatively with systemic steroids and 5-ASA. The patient tolerated her medications with no specific adverse effects, but she was not compliant with them. She is a housewife and a nonsmoker with no notable family history of related diseases.

The patient experienced an exacerbation of CD around six months prior to this admission. According to the patient history, subsequent investigation, and review of the patient's medical records, she complained of severe abdominal pain and distention with diarrhea and fever for two days. She sought medical help at an outside government hospital and was referred to our hospital accordingly. A diagnostic endoscopy was performed for this purpose and revealed findings consistent with active and severe CD, including a severely inflamed and narrowed terminal ileum, a narrowed ileocecal valve, and deep skip lesions and ulcerations throughout the colonic area that resulted in luminal narrowing. Following a biopsy, macroscopic findings included a 20-cm-long ileum and a 31 cm-long colon, thicker gut walls wrapped in fat, hemorrhagic mucosa, and an area of perforation in the ascending colon. There was no evidence of granulomas, infectious microorganisms, dysplasia, or malignancy. Microscopic features revealed distortion of the crypt architecture and transmural inflammation. The lamina propria displayed chronic inflammatory cell infiltration.

The patient had neck pain, shortness of breath, and minor chest pain a few hours following the endoscopy with a rapid development of severe abdominal pain and distention, associated with diffuse abdominal tenderness on exam. The clinical examination findings also suggested surgical emphysema in the neck and upper chest. Following an abdominal computed tomography (CT) scan, pneumoperitoneum and pneumomediastinum were discovered, raising strong suspicions of colonic perforation. An urgent laparotomy was planned. Intraoperatively, perforation of the proximal ascending colon was seen, as well as significant fat and inflammatory changes around the perforated region. Based on these findings and the endoscopic findings, the decision was taken to perform an ileocecectomy followed by an end-to-end ileocolic anastomosis. The postoperative course was uneventful with good recovery, stable vital signs, and tolerable oral intake; hence, the patient was discharged and surgery clinic follow-up was advised.

A month after her operation, the patient went into the emergency room complaining of mild abdominal pain, a feverish sensation, and a yellow discharge coming from her surgical laparotomy site. Physical examination revealed a round 2 × 2 cm, fluctuant, trans-illuminated swelling with diffuse edges at the upper part of the laparotomy scar, 1 cm below the xiphoid process that is tender, with normal overlying skin, and pus discharge. She underwent aspiration with the drainage of 15 mL of pus after abdominal ultrasonography (US) revealed signs of a subcutaneous abscess under the laparotomy scar. A culture was obtained, which gave a positive result for *Proteus mirabilis* and *Escherichia coli* (*E. coli*). She was discharged on oral antibiotics.

The patient returned to the ED after a month due to increasing pus discharge from the upper area of the laparotomy scar site, which was accompanied by no pain or fever and stable vital signs. Laboratory tests, including complete blood counts (CBC) and metabolic banal, were performed and showed high white blood cells (WBCs) (13.11 × 10^9^/L) and C-reactive protein (CRP) (26 mg/L), with all other values falling within the normal range.

**Figure 1:**
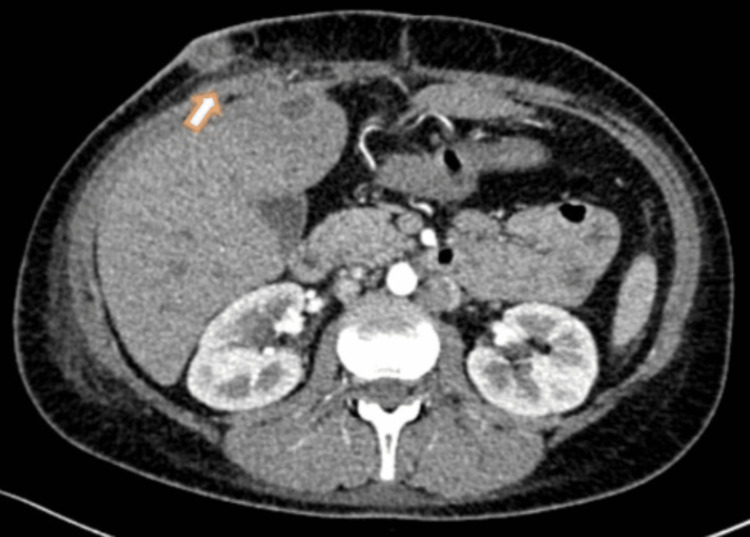
Abdominal CT scan shows an abscess anterior to segment IV of the liver, connected from one side with the ileocolic anastomosis and from the other side with a fistula tract (white arrow) that opens in turn in the anterior abdominal wall.

An abdominal CT scan with oral and intravenous contrast showed the existence of a thickened and edematous proximal transverse colon (at the site of anastomosis) and significant stranding and blurring of the surrounding fat planes and gas bubbles. These findings point to an ECF (Figure 1).

As a result, she had percutaneous CT-guided drainage of the abscess and fistula on the same day as her admission. A drain was introduced into the collection in the right upper quadrant region and subsequently replaced with a pigtail drain when it slipped from its target site.

**Figure 2:**
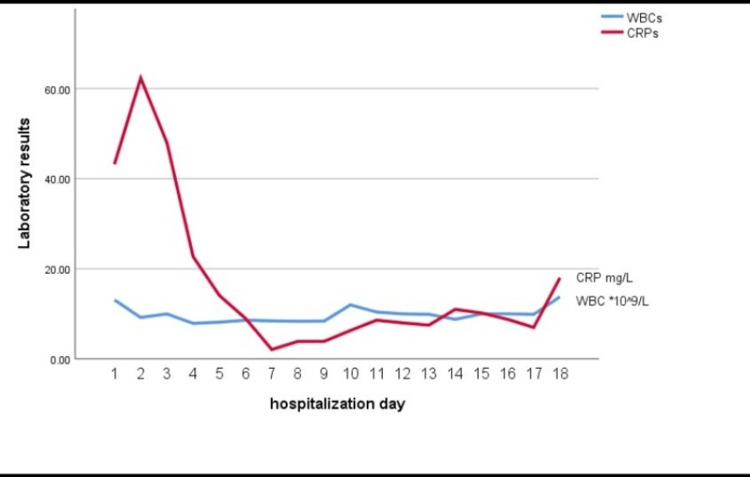
Changes in the CRP and WBCs throughout the first hospitalization. WBC: white blood cells × 10^9^/L; CRP: C-reactive protein (mg/L)

She was then maintained on oral nutrition for three days before being transitioned to an oral soft diet as tolerated. In addition, an empiric intravenous antibiotic (ceftriaxone at first and then meropenem) was administered based on the growth of the bacteria *E. coli*, *P. mirabilis*, *Enterococcus faecalis*, *Enterococcus faecium*, and *Klebsiella pneumonia* in the culture. The patient's vital signs were steady throughout her hospital stay, and her CRP dropped (from 43.2 mg/L at admission to 17.2 mg/L at discharge). Figure 2 shows the changes in the inflammatory markers throughout this admission. A subsequent abdominal CT scan with contrast revealed that the ECF had healed 10 days after the CT-guided drainage, but the fistula between the anastomosis and the reported collection had not. One day before discharge, a follow-up ultrasound (US) was taken, and it revealed a reduction in the collection size (2 cm at its greatest dimension with a drain in place). The patient was released with assurances regarding her fistula, which was thought to have spontaneous closure because she had made a considerable recovery as the fistula had no output and no signs of sepsis or local inflammation.

The patient complained of a one-week duration of general abdominal discomfort and many episodes of vomiting, fever, and chills during the current stay, which occurred about five months after the laparotomy and three months after the prior admission. A thorough physical examination was remarkable for a generalized abdominal tenderness more localized in the right upper quadrant area but no guarding or rigidity. It also revealed fluctuant swelling near the location of the previously implanted drain from the prior hospitalization in the right upper quadrant. Moreover, there was a fistulous opening in the upper portion of the midline laparotomy scar that discharged pus.

The patient was hospitalized for further assessment. WBCs were 13.0 × 10^9^/L, hemoglobin was 11.5 g/dl, CRP was 108 mg/L, erythrocyte sedimentation rate (ESR) was 120 mL/hour, blood urea nitrogen was 3.7 mmol/L, creatinine was 0.4 mg/dL, sodium was 137 mmol/L, potassium was 3.9 mmol/L, and chloride was 102.3 mmol/L according to the laboratory results at the time of admission (Table 1). There was no fever, and vital signs were stable.

**Table 1:** Initial laboratory results upon current admission.

Parameter	Result	Reference range
White blood cell count	13	4-9 k/µl
Hemoglobin	11.5	13.7-17.2 g/dL
Sodium	137	135-155 mEq/L
Potassium	3.9	3.5-4.8 mEq/L
Chloride	102.3	98-107 mEq/L
Blood urea nitrogen	3.7	5-22 mg/dL
Creatinine	0.4	0.7-1.2 mg/dL
C-reactive protein	108	0-5 mg/L
Erythrocyte sedimentation rate	120	0-15 mm/hr

Her oral intake was maintained as tolerated, intravenous (IV) antibiotics (meropenem initially) were started, a wound culture was obtained (revealing *P. mirabilis* and *E. coli*), and daily dressing and follow-up wound care were undertaken. After the gastrointestinal team reviewed her, she was reinstituted on 5-ASA and azathioprine.

An abdominal CT scan showed thickening at the transverse colon anastomosis site, along with significant stranding and blurring of the surrounding fat plan, while only modest dilation was visible at the small bowel anastomosis site. Anastomosis was connected by a fistula tract to a collection measuring 2.1 × 1.7 cm craniocaudal × transverse × anteroposterior, indenting the anterior surface of the segment IV of the liver with edema of the surrounding hepatic parenchyma. The collection in turn was opened in the anterior abdominal wall via at least two openings, one of which was ended by a subcutaneous collection measuring 1.8 × 1.4 cm at the upper part of the laparotomy scar. Another smaller collection, spanning around 1 × 0.6 cm in the cross-section and located in the subhepatic area, was linked to the anterior hepatic collection that was previously noted.

On the third day after admission, the right upper quadrant abscess spontaneously burst, CRP dropped to 40.42 mg/L (from 108 mg/L), and WBC to 9.42 10^9^/L (from 13.0× 10^9^/L). Following that, an abdominal CT was performed for evaluation, and the results were the same as the previous CT, with the exception of a decrease in the collection size.

The patient was advised about alternative care choices, but she insisted that any operative management with an abdominal scar like the laparotomy one and recurrent dressings be totally refused. She also asked for definitive management, with an almost nil likelihood of recurrence.

A multidisciplinary team (MDT) meeting, attended by the general surgery team, gastroenterology team, radiology team, and infectious specialist, opted to embolize this fistula with fibrin glue injection and coiling.

**Figure 3:**
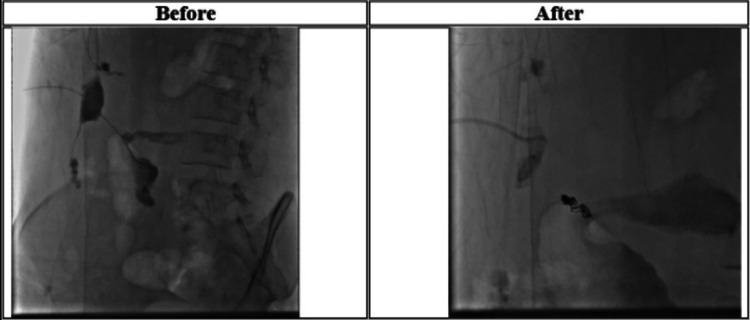
Fistulogram study revealing the successful cannulation of the fistula tract until the site of anastomosis, followed by injection of glue (before and after).

WBC was 9.42 10^9^/L and CRP was 40.40 mg/L on the fourth day after admission. The procedure involved a selective puncture of the skin defect, followed by fistulography, which revealed a subcutaneous fistula tract that extended to both the abscess collection in the right upper quadrant and the end of the ileocolic anastomosis. Cannulation of the fistula tract was attempted down to the anastomosis region, followed by coiling and the injection of 1 mL of glue. After successfully eliminating the fistula tract, the catheter was pulled back, as demonstrated by a selective fistulogram. In addition, a pigtail catheter was used to drain the abscess, followed by the injection of 0.5 mL of glue into the abscess wall (Figure 3).

**Figure 4:**
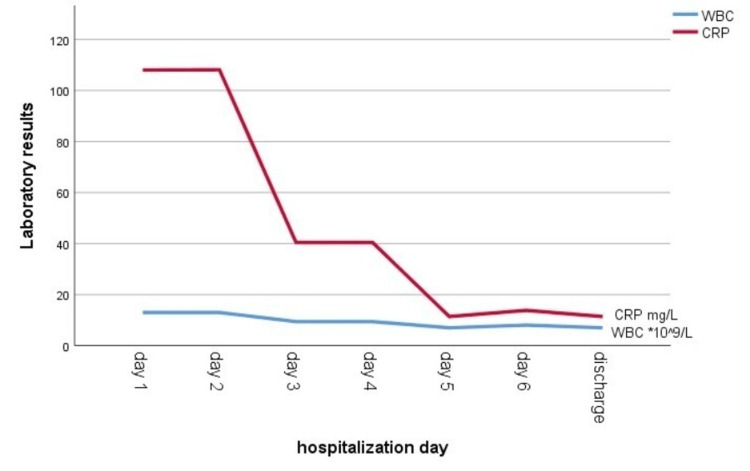
Changes in the inflammatory marker (CRP) and white blood cells (WBCs) throughout the second hospitalization.

Over the course of the next few days, the patient remained stable with no complaints or complications; CRP was 11.4 mg/L and WBC was 7 × 10^9^/L. Figure 4 shows the variations in inflammatory markers during this hospitalization. The abdominal examination after the procedure was soft with no tenderness. There was a dry dressing over her abdominal openings, which were almost closed with no discharge. Following an abdominal CT scan, there was a streak artifact at the site of the fistulous tract, mostly due to coiling; the superficial collection was almost drained; and the previously mentioned deep collection substantially decreased in size.

**Figure 5:**
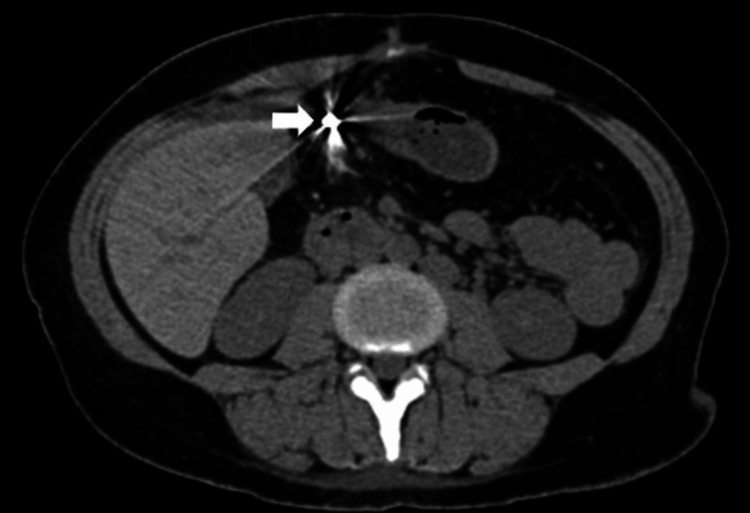
Abdominal CT image demonstrating the apparent coil (white arrow) with almost absence of the collections.

These results were satisfying to the involved team; therefore, the patient's diet was gradually upgraded with careful monitoring and follow-up, and she stayed on antibiotics that were adjusted from meropenem to piperacillin-tazobactam according to culture and sensitivity. Three days later, another CT scan was done prior to discharge and showed complete drainage of the superficial abscess and a decrease in the size of the deep abscess (Figure 5). The patient was sent home with oral antibiotics (metronidazole and ciprofloxacin); starting infliximab treatment was also advised by her gastroenterologist, who was continuing the patient's follow-up nearly every month. Long-term follow-up has been attempted in the gastroenterology and surgery clinics. She continued to remain stable, with no recurrence of her fistula. It is noteworthy to mention that the patient had a follow-up period of about one year in the gastroenterology clinic, during which time there were no confirmed recurrences. Table 2 provides an overview of the imaging data collected throughout the patient's hospitalization.

**Table 2:** Summary of the imaging data

Imaging	Findings
CT scan (five months prior to admission, after colonoscopy and iatrogenic perforation)	Pneumoperitoneum and pneumomediastinum, with high suspicion of colonic perforation
Abdominal ultrasound (four months prior to admission)	Evidence of a subcutaneous abscess beneath the laparotomy scar
Abdominal CT scan with oral and intravenous contrast (four months prior to admission)	There is a thickened and edematous proximal transverse colon (at the site of anastomosis) with severe stranding and blurring of the adjacent fat planes and gas bubbles. This is connected with a tubular collection indenting the anterior surface of the segment IV of the liver, containing fluid and gas, which in turn connect to the anterior abdominal wall and open via at least two openings at the upper portion of the laparotomy scar. This finding suggests an enterocutaneous fistula (Figure 1).
Abdominal CT scan with contrast (10 days after the percutaneous drainage)	The enterocutaneous fistula closed, while the fistula between the anastomosis and the collection remained present.
Abdominal CT scan (upon index admission)	There is a thickening at the transverse colon site of the anastomosis. The anastomosis is connected by a fistula tract to a collection, which in turn opens in the anterior abdominal wall via at least two openings; one of them is ended by a subcutaneous collection measuring 1.8 x 1.4 cm at the upper part of the laparotomy scar.
Follow-up abdominal CT scan after coiling	There is a streak artifact at the site of the fistulous tract, mostly due to coiling; the superficial collection was almost drained, and the previously mentioned deep collection substantially decreased in size (Figure 3).
Abdominal CT scan upon discharge	There is complete drainage of the superficial abscess and a decrease in the size of the deep abscess.

It is worth noting that all of the interventions, in this case, were carried out by expert physicians, all of whom were listed in the author's section.

Other considerations

The patient declined to have any surgery because she had a history of depression after her previous laparotomy. This choice, therefore, has an impact on the management strategy. This study shows that depression before surgery did not affect the outcome [10]. Engaging patients in decision-making improves their satisfaction regarding the care they receive, as they become more adherent to be compliant with the treatment and follow-up, and decreases anxiety and psychological stresses that they may experience [11].

The patient had financial concerns, which made the diagnosis a challenge; thus, the majority of the imaging and laboratory testing was done outdoors in a government facility. As each facility's tests and images have their own medical systems and our hospital is unable to access them, some data were lost throughout the lengthy process of gathering them. Due to her financial difficulties, the patient chose to use her insurance for treatment at less specialized government facilities rather than comply with the follow-up at our hospital.

The patient stated that she was happy with this choice because embolization spared her from surgery or any scars.

## Discussion

An ECF is an extrinsic fistula formed between the gastrointestinal organs and the skin. It might be categorized according to the pathophysiology, organ involved, output volume, or tract length. This classification gained its significance through its use as a reference for predicting the fistula's favorability to close spontaneously and the subsequent outcomes [5]. Fistulas are either iatrogenic (85%) or spontaneous (about 15% in the sittings of IBDs, radiation, diverticular diseases, appendicitis, ischemic bowels, and some infectious etiologies ) [12]. The different subcategories and many predictive factors led to significant variations in the mortality rates (5.5-33%), with sepsis remaining the leading cause [13].

CD has been shown to be one of the risk factors for fistula formation, with high recurrence rates. According to Schwartz et al., 35% of CD patients experience at least one fistula over the duration of their illness, with 33% taking place in the first 10 years and 50% in the next decade [14]. It is important to highlight that CD-associated fistulas occur either spontaneously in the diseased bowel with unclear pathophysiology, which might be related to the sustained transmural inflammation and subsequent transition of the polarized epithelial cells to migratory invasive mesenchymal cells, resulting in remodeling and fistula formation [2], or post-surgically, mostly at the anastomotic site following bowel resection, thus being considered a postsurgical complication [3]. CD patients are highly susceptible to undergoing many procedures. A systemic review by Frolkis et al. showed that about 21% of CD patients will at least undergo one surgical intervention within the first 10 years of their disease onset [15], increasing the likelihood of ECFs. We postulate in our case that the patient's history of CD contributed to the development of the reported fistula and the subsequent delay in its healing.

Iatrogenic bowel perforation during colonoscopy is an uncommon event with serious consequences and a 25% mortality rate. Several risk factors have been addressed. Some of them include having a diseased bowel, such as CD, being old, being female, having a therapeutic colonoscopy rather than a diagnostic one, and having poorly trained physicians.

Iatrogenic colonoscopy perforation might manifest with immediate or delayed symptoms, depending on the site and severity of the perforation. ICP management is tailored to each patient's unique situation and may be conservative, endoscopic, or surgical, with the potential for various surgical techniques [16]. Weng et al. reported a case in which a CD patient had perforation following an endoscopy and was treated surgically with bowel resection at the site of perforation due to the instability exhibited in the patient's status and the extensive complications following ICP [17].

In our case, a diagnostic endoscopy revealed evidence of a highly inflamed, narrowed terminal ileum and a narrowed ileocecal valve compatible with a severe CD flare. Hours later, the patient had pneumoperitoneum and pneumomediastinum. There was a perforation of the proximal ascending colon with significant fatty and inflammatory changes around the perforated region. According to this and the microscopic and gross findings at the time of the laparotomy, it was decided to resect the diseased intestine where the perforation was also discovered (the ileocecal region) with anastomosis.

Fistulas are associated with intraabdominal and cutaneous abscesses in about 40% of cases. Abscess-fistula complex etiologies resemble those for fistula formation. Iatrogenic causes following surgical interventions are the leading etiologies. The suggested pathophysiology by which cutaneous abscesses develop is that the leakage of bowel content will either diffuse and result in peritonitis or localize, forming a localized abscess. These abscesses will continue to spread, seeking the least resistant site, which is usually the surgical site [18]. Furthermore, it was reported that in the presence of a cutaneous abscess, the fistula may not be identified unless the associated abscess is drained and the cavity shrinks [19].

A fistula diagnosis is a combination of clinical evaluation, laboratory testing, and radiological imaging. Fistulas can have a variety of clinical manifestations, including nonspecific symptoms, such as fever, chills, weakness, malaise, and leukocytosis. In the post-surgical acute period, these symptoms are typically severe, and in some circumstances, they might be indicators of sepsis or other life-threatening consequences. More localized manifestations are directly related to the site of the fistula and the involved organ. Abdominal pain, surgical site discharge of the bowel content, diarrhea, and skin irritation are the most common symptoms of ECFs [20].

In this case, the patient initially presented complaining of abdominal pain, fever, and fluctuant swelling at the upper part of the laparotomy scar with pus discharge. Abdominal US showed evidence of a subcutaneous abscess beneath the laparotomy scar, for which she underwent aspiration and drainage in the ED and was discharged home. Shortly after, the patient came back with an increasing discharge from the same surgical site. She was subsequently diagnosed with an ECF, raising the possibility that this fistula was not apparent at the first presentation due to the abscess formation. Furthermore, the patient had an intraabdominal abscess along with the prescribed fistula at the following admissions. 

Laboratory workups include CBC for WBC and HGB assessment; a metabolic panel to evaluate possible fluid and electrolyte abnormalities, especially in high-output fistulas; and a lactate level to assess bowel ischemia [20]. Radiological imaging is important for confirming the diagnosis, locating the anatomy, identifying any associated abnormalities, including the presence of abscesses, and planning the management. For these purposes, many modalities have been used and addressed in the literature. US, fistulogram, small bowel studies, cross-sectional images (CT and magnetic resonance imaging (MRI)), and endoscopy are all used, and each has its own advantages and limitations in ECF diagnosis [21]. A CT scan is considered the best option in the initial evaluation of ECFs, as it has high sensitivity in addressing the exact anatomy of ECFs and associated abscesses or possible distal obstructions and foreign bodies [22]; however, the risk of radiation is still a problem. MRI as an alternative option in ECF diagnosis, especially in the presence of concomitant CD, has also been suggested in the literature, as it has been used successfully in perianal fistula diagnosis. This benefit has been supported by the absence of radiation exposure risk and the ability to identify and differentiate inflammation and stenosis intra- and extra-intestinally [23].

The fistulogram was the preferred technique in diagnosing ECFs, but nowadays, it is used in conjunction with other modalities as it lacks the ability to define the fistula’s anatomic location and the nearby bowel conditions below and above the fistula. In addition, the contrast's diagnostic use is constrained by the presence of abscesses or other abnormalities, such as edema and debris, that prevent the contrast from reaching the intestine lumen [21]. The use of another radiological modality is essential as well because US has been demonstrated to have a number of drawbacks, including the necessity of a skilled operator, the difficulty of seeing the duodenum and jejunum, and obesity [24].

In this case, US imaging confirmed the initial diagnosis of a cutaneous abscess; after the patient was admitted, a CT scan was taken, which revealed an ECF tract extended to the previously prescribed anastomosis and an intra-abdominal abscess ahead of segment IV of the liver. A CT scan was used again when the fistula failed to spontaneously close and revealed the same fistula tract with abscess collections in the right upper quadrant of the abdomen and subhepatically. A fistulogram was also used in this case when definitive intervention was attempted; it confirmed the CT findings regarding fistula tract extension and the elimination of this tract after the intervention.

Treating ECFs poses a high challenge, and the need for a multidisciplinary decision is mandatory. The multidisciplinary team should consist of a surgeon, a gastroenterologist, an infectious specialists, an interventional radiologist, and trained nurses and therapists [7]. In this case, the surgical team has been involved in the case since the patient's admission with regard to potential surgical intervention. In addition to the infectious team, which was contacted with a suitable choice of antibiotic regimens, the gastrointestinal team was also involved in her condition and her follow-up, as was the radiological team, which carried out the therapy.

Medical management has a low success rate (less than 30%) but remains the initial step in the management of ECFs, even for those who will proceed to surgical options. This includes stabilizing the patient with proper resuscitation and electrolyte replenishment, controlling infection, providing nutritional support, and providing wound care. The limited success ability with this management is seen mostly in cases of high output, short fistulas, and fistulas in unfavorable sites [12]. Even though surgical closure rates are up to 85%, surgical options still have high complications and recurrence possibilities. This highlights the need for alternative options to override the surgical route [6]. Interventional radiology has played a major role in this area, with the main use being draining associated abscesses and any collection, therefore speeding up the closure process of the fistula and preventing further infections [25].

Fibrin glue with endoscopic control has been used as an alternative to surgery when an ECF fails to close with conservative management to decrease the expected closure time. This option's success requires specific features in the treated fistula. Low-output and long-tract fistulas showed to be more favorable to close using a fibrin sealant [6]. In a case-control study on 23 patients treated with fibrin glue, González et al. reported that the complete closure time following fibrin glue application was eight ± four days in the study group versus 19.1 ± six days in the control group [26].

Fibrin glue is a topical tissue adhesive that may be used in any surgical specialty, such as to stop bleeding after an organ injury. It possesses hemostatic and adhesive properties. Its value is particularly well established in the disciplines of thoracic surgery, ear, nose, and throat (ENT) surgery, cardiovascular surgery, and neurosurgery. Fibrin glue has been widely used to treat anal fistulas. Fibrin glue produced immediate hemostasis, good healing of bleeding sites, no secondary bleeding, and no inflammation. Adverse events include local swelling, pain, and slow healing of the bleeding site. An anaphylactic reaction has been reported when using fibrin glue [27]. 

Another alternative option is treatment with platinum coils and embolization, which showed high effectiveness and low morbidity. Therefore, it should be considered a therapeutic option in cases of persistent ECFs despite medical management and even those difficult to manage surgically [9].

To our knowledge, no previous studies have reported the use of both fibrin glue and coiling as a combination in treating ECFs. However, after conservative therapy and percutaneous catheter drainage of pancreatic abscesses failed to repair pancreatoduodenal fistulas, the combination of coil embolization and the application of fibrin glue was reported in a case study as an effective way to achieve closure [28].

In our case, the patient refused any surgical intervention as the laparotomy was traumatic to her, and she requested a fast and effective treatment with minimal recurrence risk. The patient's history of CD with fulminant recurrent exacerbations made the surgical intervention less favorable in managing her ECF due to the higher recurrence chances. With the help of a multidisciplinary team, the choice was made to apply two approved options (fibrin glue and coiling) simultaneously, which were used in the literature separately in treating ECFs, to maximize their efficacy and obtain a full recovery.

In conclusion, an ECF is a serious complication that arises from different etiologies, and its management requires multidisciplinary teamwork. Medical conservative management is the initial step in all ECF cases. For an ECF that fails to close with conservative management, other options should be considered; although surgical management is the definitive option, complications could occur. Interventional radiology, fibrin glue injection, and embolization with coiling are other plausible modalities that have gained significant attention as alternative options.

To our knowledge, this is the first case to assess the efficacy and safety of using both fibrin glue injection and coiling as novel management for ECFs to obtain a definitive fistula closure. This option should be taken into consideration as an alternative to surgical management that could predispose the patient to the risk of fistula recurrence and many other surgical complications, including recurrence, infections, scarring, abscess formation, dehiscence, short bowel syndrome, and leakage.

## Conclusions

An ECF is an abnormal connection between any of the gastrointestinal parts and the skin; it can be brought on by iatrogenic causes or spontaneously occur in the setting of different diseases that affect the gastrointestinal tract. This abnormality could lead to serious complications and even death. As a result, care should be focused on treating any related potential comorbidities before focusing on fistula closure.

In this patient, a persistent fistula was managed by embolization using fibrin glue and coiling. This management should be taken into consideration as an effective alternative option for surgical management owing to its simplicity and low mortality. However, this is a single successful case, and the need for further controlled studies is important to confirm the effectiveness of this option. This case report could serve as evidence to support the implementation of such studies.

## References

[1] Gecse K, Khanna R, Stoker J, Jenkins JT, Gabe S, Hahnloser D, D'Haens G (2013). Fistulizing Crohn’s disease: diagnosis and management. United European Gastroenterol J.

[2] McGregor CG, Tandon R, Simmons A (2023). Pathogenesis of fistulating Crohn's disease: a review. Cell Mol Gastroenterol Hepatol.

[3] Poritz LS, Gagliano GA, McLeod RS, MacRae H, Cohen Z (2004). Surgical management of entero and colocutaneous fistulae in Crohn's disease: 17 year's experience. Int J Colorectal Dis.

[4] Mawdsley JE, Hollington P, Bassett P, Windsor AJ, Forbes A, Gabe SM (2008). An analysis of predictive factors for healing and mortality in patients with enterocutaneous fistulas. Aliment Pharmacol Ther.

[5] Evenson AR, Fischer JE (2006). Current management of enterocutaneous fistula. J Gastrointest Surg.

[6] Owen RM, Love TP, Perez SD (2013). Definitive surgical treatment of enterocutaneous fistula: outcomes of a 23-year experience. JAMA Surg.

[7] Orangio GR (2010). Enterocutaneous fistula: medical and surgical management including patients with Crohn's disease. Clin Colon Rectal Surg.

[8] Olayode A, Kizer R (2012). Fibrin glue treatment of enterocutaneous fistula in Crohn’s disease: 1290. Am J Gastroenterol.

[9] Andrés Moreno AM, Ponce Dorrego MD, Jiménez Gómez J, Gómez Cervantes M, Vilanova Sánchez A, López Gutiérrez JC, López Santamaría M (2021). Combined treatment of enterocutaneous fistula with laser diode and embolization. Cir Pediatr.

[10] Maroof H, Van Chi Mai D, El-Kafsi J, De'Ath HD (2023). The impact of depression in patients undergoing emergency abdominal surgery: an exploratory study. World J Surg.

[11] Krist AH, Tong ST, Aycock RA, Longo DR (2017). Engaging patients in decision-making and behavior change to promote prevention. Inf Serv Use.

[12] Rahman FN, Stavas JM (2015). Interventional radiologic management and treatment of enterocutaneous fistulae. J Vasc Interv Radiol.

[13] Gribovskaja-Rupp I, Melton GB (2016). Enterocutaneous fistula: proven strategies and updates. Clin Colon Rectal Surg.

[14] Schwartz DA, Loftus EV Jr, Tremaine WJ, Panaccione R, Harmsen WS, Zinsmeister AR, Sandborn WJ (2002). The natural history of fistulizing Crohn's disease in Olmsted County, Minnesota. Gastroenterology.

[15] Frolkis AD, Lipton DS, Fiest KM (2014). Cumulative incidence of second intestinal resection in Crohn's disease: a systematic review and meta-analysis of population-based studies. Am J Gastroenterol.

[16] de'Angelis N, Di Saverio S, Chiara O (2018). 2017 WSES guidelines for the management of iatrogenic colonoscopy perforation. World J Emerg Surg.

[17] Weng E, Valencia DN, Krudy ZA, Ali M (2020). Intraperitoneal and extraperitoneal colonic perforation following diagnostic and therapeutic colonoscopy with crohn's-related stricture dilation. Cureus.

[18] Ballard DH, Erickson AE, Ahuja C, Vea R, Sangster GP, D'Agostino HB (2018). Percutaneous management of enterocutaneous fistulae and abscess-fistula complexes. Dig Dis Interv.

[19] Ballard DH, Hamidian Jahromi A, Li AY, Vea R, Ahuja C, D'Agostino HB (2015). Abscess-fistula complexes: a systematic approach for percutaneous catheter management. J Vasc Interv Radiol.

[20] Tuma F, Crespi Z, Wolff CJ, Daniel DT, Nassar AK (2020). Enterocutaneous fistula: a simplified clinical approach. Cureus.

[21] Lee JK, Stein SL (2010). Radiographic and endoscopic diagnosis and treatment of enterocutaneous fistulas. Clin Colon Rectal Surg.

[22] Hollington P, Mawdsley J, Lim W, Gabe SM, Forbes A, Windsor AJ (2004). An 11-year experience of enterocutaneous fistula. Br J Surg.

[23] Fidler J (2007). MR imaging of the small bowel. Radiol Clin North Am.

[24] Kwon SH, Oh JH, Kim HJ, Park SJ, Park HC (2008). Interventional management of gastrointestinal fistulas. Korean J Radiol.

[25] Taggarshe D, Bakston D, Jacobs M, McKendrick A, Mittal VK (2010). Management of enterocutaneous fistulae: a 10 years experience. World J Gastrointest Surg.

[26] Avalos-González J, Portilla-deBuen E, Leal-Cortés CA (2010). Reduction of the closure time of postoperative enterocutaneous fistulas with fibrin sealant. World J Gastroenterol.

[27] (2016). Fibrin glue. Meyler’s Side Effects of Drugs (Sixteenth Edition).

[28] Choi KM, Kim YD, Ahn JH (2013). Closure of pancreatoduodenal fistula using vascular occluding coil embolization and fibrin glue injection: a case study. Korean J Hepatobiliary Pancreat Surg.

